# “The SOFTVETS Competence Model” – a preliminary project report

**DOI:** 10.3205/zma001446

**Published:** 2021-03-15

**Authors:** Christin Kleinsorgen, Evelyn Steinberg, Rudolf Dömötör, Jelka Zabavnik Piano, Jože Rugelj, Mira Mandoki, Lada Radin

**Affiliations:** 1 University of Veterinary Medicine Hannover, Foundation, ZELDA - Centre for E-Learning, Didactics and Educational Research, E-Learning-Department, Hannover, Germany; 2 University of Veterinary Medicine Vienna, Office of the Vice-Rectorate for Study Affairs, Vienna, Austria; 3 Vienna University of Economics and Business, Vienna, Austria; 4 University of Ljubljana, Veterinary Faculty, Ljubljana, Slovenia; 5 University of Ljubljana, Faculty of Education, Ljubljana, Slovenia; 6 University of Veterinary Medicine Budapest, Budapest, Hungary; 7 University of Zagreb, Faculty of Veterinary Medicine, Zagreb, Croatia

**Keywords:** veterinary medicine education, communication, entrepreneurship, digital competences

## Abstract

**Aim:** Recent developments measured using statistics and surveys among veterinarians show that integrating key competence training into veterinary education is becoming increasingly important.

This article describes the collaborative development process of the first work package within the SOFTVETS project. The SOFTVETS project aims to create a competence model and an ideal version of a soft skills curriculum that can be implemented in veterinary higher education throughout Europe.

**Method:** In the course of a desk research phase, a literature review and an inventory of the current practice of key competence training within veterinary education was carried out. An initial set of recommendations for three competence areas was developed using the Handbook for Internal Quality Management in Competence-Based Higher Education. Finally, an alternating sequence of individual and collaborative expert reviews was carried out.

**Result:** Experts from five European countries participated in the process. The derived competence model consisted of the following three competence areas with the corresponding number of defined competences: ten communication, nine entrepreneurial and eight digital competences.

**Conclusion:** In the next work packages, learning objectives, teaching and assessment methods will be collected. Training concepts for facilitators to provide professional competence training will be established. In addition, an evaluation toolkit will be developed to standardise the implementation, evaluation and assessment of competence training events.

The SOFTVETS competence model should help educators to be able to integrate the training of key competence training into the veterinary curriculum. This detailed list of competences can also be used as a tool to identify existing deficiencies and thus enable further curricular changes.

## Zusammenfassung

**Ziel: **Jüngste Entwicklungen, die anhand von Statistiken und Umfragen unter Veterinärmediziner*innen ermittelt wurden, zeigen, dass die Integration von Schlüsselkompetenztraining in die veterinärmedizinische Ausbildung immer wichtiger wird.

Dieser Artikel beschreibt den gemeinschaftlichen Entwicklungsprozess des ersten Arbeitspakets im Rahmen des SOFTVETS Projekts. Das SOFTSVETS Projekt hat sich zum Ziel gesetzt ein Kompetenzmodell und ein Idealbild eines Soft-Skills-Curriculums zu erschaffen, welches in veterinärmedizinischen Ausbildungsstätten in Europa eingesetzt werden kann.

**Methode: **Während der Schreibtischforschungsphase wurden eine Literaturrecherche und eine Inventur der aktuellen Praxis für Schlüsselkompetenztraining im Veterinärmedizinstudium durchgeführt. Initial wurden auf Basis des Handbuchs für Internes Qualitätsmanagement in Kompetenz-basierter Hochschulausbildung Empfehlungen für drei Kompetenzbereiche entwickelt. Anschließend wurden abwechselnd individuelle und gemeinschaftliche Expertenbewertungen vorgenommen.

**Ergebnisse:** Expert*innen aus fünf europäischen Ländern waren am Prozess beteiligt. Das erarbeitete Kompetenzmodell besteht aus den folgenden drei Kompetenzfeldern mit der dazugehörigen Anzahl von definierten Kompetenzen: 10 kommunikative, 9 unternehmerische und 8 digitale Kompetenzen.

**Fazit: **In den nächsten Arbeitspaketen werden Lernziele sowie Lehr- und Prüfungsmethoden gesammelt. Es werden Schulungskonzepte für Lehrkräfte zur Vermittlung von Fachkompetenztraining festgelegt. Außerdem wird ein Evaluations-Toolkit entwickelt, um die Implementierung, Evaluation und Bewertung der Lehrveranstaltungen zu standardisieren.

Das SOFTVETS Kompetenzmodell soll Lehrpersonal helfen das Training für Schlüsselkompetenztraining in veterinärmedizinische Curricula zu integrieren. Die detaillierte Kompetenzliste kann auch als Hilfsmittel zur Identifizierung von bestehenden Defiziten und der Weiterentwicklung von Curricula verwendet werden.

## Introduction

Key competences are an important element in the training of veterinarians and the acquisition of these should not be left to chance. From the perspective of practising veterinarians, educators, the accreditation authority for veterinary education establishments within Europe and the students themselves, the need for didactic concepts for integrating the teaching of key competences is increasing. The acquisition and further development of knowledge, skills and attitudes are essential for the veterinary practice and constant modernisation of the profession. Current developments in the labour market also offer versatile new opportunities for veterinarians [1]. Therefore, training should and must address these aforementioned aspects in order to provide veterinarians with new forms of flexibility and job security within their profession. Recent research and surveys amongst veterinarians have shown an increasing need for integrating life skills training in veterinary education [2]. Skill acquisition and development are essential for the performance and modernisation of the profession [1], [2]. The European Association of Establishments for Veterinary Education (EAEVE) as accreditation authority for veterinary education establishments also requests proof of competence training within their visitation and has provided a list of Day-One-Competences ([3], Annex 2). As there are several definitions for the term competence, we refer to that given by the veterinary accreditation body: “Competence is a concept that integrates knowledge, skills and attitudes. Competence requires acquisition of technical skills but further involves applying relevant knowledge, and having the confidence and ability to transfer what has been learnt to a variety of contexts.”([3], p.29).

Within competence-based (veterinary) medical education [4], [5], several main topics are covered, often referred to as areas or domains of competence [6]. The competence-based veterinary education [7] covers areas such as animal care for individuals, herd management and public health. So-called non-technical professional competences cover various areas such as self-competence, social and communication competences, business management, entrepreneurship, media, and digital literacy [8]. In particular, the non-technical professional competence training currently varies greatly within the veterinary curricula, even if there is a standardised evaluation within European institutions [3]. Since there are already efforts to develop curricula in the field of public health and animal welfare [9] or business management [10], [11], and the demand at the participating institutions was particularly pronounced, our research focus was on the following three key competences: communication, entrepreneurship and digital skills.

Communication is a core clinical skill [12]. Furthermore, communication is a set of learned skills that needs to be taught [13]. The quality of communication between veterinarian and patient owner is of great importance for client’s loyalty [14] and satisfaction [15], but also for the outcome of the consultation [16]. Lack of communicative competence on the part of the veterinarian is a common cause of complaints and errors [17]. Therefore, teaching, learning and assessing communication competence within veterinary undergraduate studies is essential [18].

The European Commission identified “sense of initiative and entrepreneurship” as one of the eight key competences necessary for European citizens [19]. Similar to communication competences, “there is a growing awareness that entrepreneurial skills, knowledge and attitudes can be learned and in turn lead to the widespread development of entrepreneurial mind-sets and culture, which benefit individuals and society as a whole” ([20], p.5). Furthermore, “EntreComp defines entrepreneurship as a transversal competence, which applies to all spheres of life: from nurturing personal development, to actively participating in society, to (re)entering the job market as an employee or as a self-employed person, and also to starting up ventures (cultural, social or commercial).” ([20], p.6). Entrepreneurial skills are also seen as essential competences within the veterinary profession [5] and are identified as an indicator of career success [21]. They include a set of cognitive and practical skills applied individually or within a group setting. Employability, as well as being able to recruit employees in their future career are relevant for many veterinary graduates [22]. Furthermore, veterinarians are often involved in research and innovation, which leads to new technologies and products. There are many competences that can be summarised within the area of entrepreneurship that are relevant for the veterinary profession. Hence, these should be addressed within veterinary education [22]. First approaches to integrating these skills into undergraduate veterinary education have already been made [10], [11].

Digitisation affects all professions and does not stop at the health care professions, so that reforms are necessary [23]. Digital literacy and media competence are not only important in scientific work, but are relevant for everyone at work, at home, in their capacity as citizens and as consumers. The European Commission defined that “digital competence involves the confident and critical use of Information Society Technology (IST) for work, leisure and communication. It is underpinned by basic skills in information and communication technology (ICT): the use of computers to retrieve, assess, store, produce, present and exchange information, and to communicate and participate in collaborative networks via the Internet.” ([19], p.L394/15–16). Higher salaries and better employability are associated with more frequent use of information and communication technology in the workplace [24]. Similar to entrepreneurship, digital skills are therefore also relevant for the veterinary profession in order to remain competitive on the market and to be able to offer modern and optimal care services. As far as we know, the use and application of technology in veterinary education has increased significantly [1], [25], [26]. The European Commission published “The Digital Competence Framework for Citizens” (DigComp) [27], [28], which includes general competences that every citizen should acquire. Yet, there is still no broad competence model for digital skills training in undergraduate veterinary education. Therefore, it is essential to integrate digital competence training in higher education. 

Despite the fact that some frameworks already exist, only a few directly applicable competence models are available. Thus, a consortium was set up to establish a pan-European soft skills curriculum for undergraduate veterinary education (SOFTVETS project). The SOFTVETS project aims to create a competence model by producing an ideal version of a soft skills curriculum that could be applicable in veterinary higher education throughout Europe.

The SOFTVETS Competence model shall adhere to the Day One Competences [3], which are in agreement with the EU Directives, Regulations and Proposals related to a veterinary professional qualification. Furthermore, the Handbook for Internal Quality Management in Competence-Based Higher Education (IQM-HE-Handbook) [29] shall be used to develop the SOFTVETS Competence model.

This article describes the collaborative development process of the first work package within the SOFTVETS project and presents the results.

## Project description

In the course of a desk-research-phase, a literature review as well as inventory of the current practice of key competence training in veterinary education were performed. For the inventory of practices, self-evaluation reports of European veterinary institutions from the accreditation throughout EAEVE, literature as well as published curricula were searched for elements regarding communication, entrepreneurial and digital skills training. After this desk research phase, an initial set of recommendations for the three competence areas was developed using the IQM-HE-Handbook [29]. The following five quality criteria were addressed to define and revise the initial theoretical competence model:

1. abstract competence definition broader than learning outcomes, but specifically for the field of study
2. components of knowledge and skills included within competences
3. competences organised in separate areas
4. definition of competence levels for knowledge and skills
5. competence development addressing significant study cornerstones (e.g. end of term, degree) [30].

This first draft included 12 communication competences, 21 digital competences and 15 entrepreneurial competences. It was sent to experts from the project member institutions for review. An alternating sequence of individual reviews by experts and online discussions was conducted. During the first online conference, the terminology for the definition of competences according to the IQM-HE-Handbook was introduced. In addition, some results from the desk research phase were presented to provide contextual and background information on the first draft.

Within each of the three competence areas, two online experts’ discussions were held. Within the first and second online meetings, the following questions were discussed:

- Which competences should veterinary students have acquired by the end of their education?
- Which competences do you teach already and how?
- Are any competences missing?
- Can any competences be deleted?
- Are the definitions of competences accurate and specific enough?
- How many ECTS can and should be accredited?

**Figure 1:**
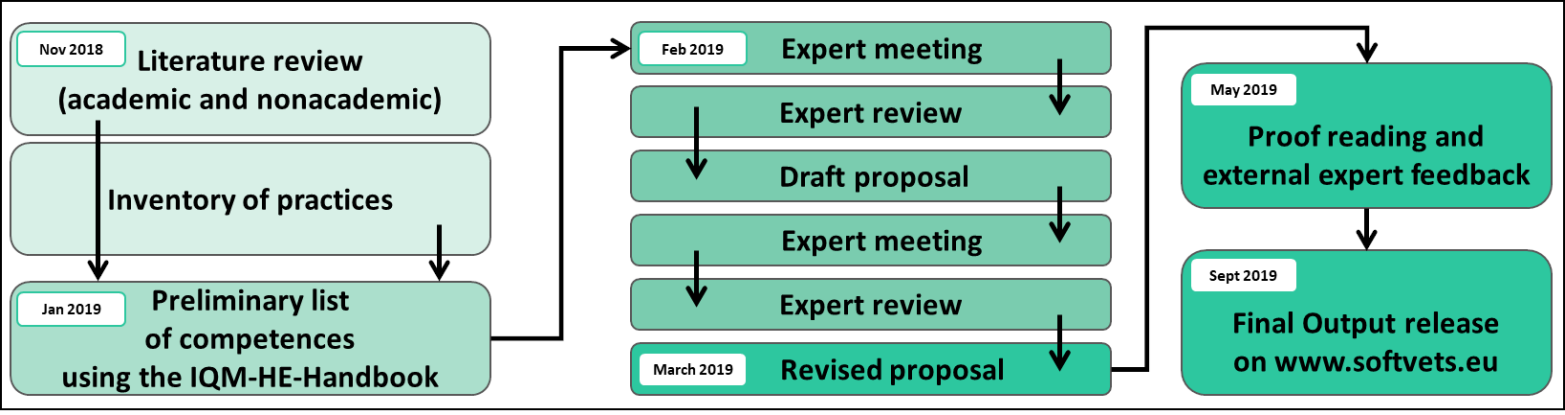
Collaborative process of development of SOFTVETS competence model

Experts from five European countries participated in the reviewing process. Experts were chosen and recruited on a voluntary basis by each institution. Within each expert group veterinarians involved in competence training at the university as well as non-veterinary educators for the professional competence area within veterinary and non-veterinary education were included in the project. Eleven experts from the field of communication were involved. Five experts in digital literacy and five experts in entrepreneurship participated. The collaborative process is displayed in figure 1 (Fig. 1). During the review process, the list of competences was further developed by exclusion, distinction and refinement.

Finally, the document was sent to experts for proofreading and a review of the content. The final document was approved by the Standing Committee on Veterinary Education (SCoVE) of the International Veterinary Student Association (IVSA) and the Accreditation Authority for Veterinary Education Establishments within the Europe European Association of Establishments for Veterinary Education (EAEVE).

## Results

For each competence, a number, a long description and a short name were defined. Each competence addressed cognitive and practical aspects. In addition, the level of proficiency (Foundation, Intermediate, Advanced, Expert) that students should reach by the end of a SOFTVETS training or by the end of their studies was agreed upon. As the SOFTVETS competence training may be included as a whole course, or it could be integrated within other subjects throughout the undergraduate study programme, the levels of proficiency were differentiated. Furthermore, students may gain in their level of proficiency in each competence during further instructional or extracurricular training, including internships. Therefore, an expected level of proficiency to be attained by the end of the undergraduate veterinary studies programme was defined.

The SOFTVETS Competence model is structured into the following three competence areas:

- Communication Competences (see table 1 (Tab. 1))
- Entrepreneurial Competences (see table 2 (Tab. 2))
- Digital Competences (see table 3 (Tab. 3)).

The authors assumed that the three competence areas are tightly integrated. The overall 27 competences are also interrelated and interconnected and as a result should be treated as parts of a whole. We are not suggesting that the learner should acquire the highest level of proficiency in all competences, or have the same proficiency for all the competences.

Within this model, competences have two aspects: a cognitive aspect (knowledge) and a practical one (skill). For each competence and for each aspect of a competence, a level is defined that students should have acquired by the end of the study programme. 

### 3.1. Communication competences

For the teaching, learning and assessment of communication skills in (veterinary) medical education, several frameworks from North America [31], [32], [33], the United Kingdom [34], [35] and the EU [36], [37], [38], [39] already exist. In the first draft, the competence areas understanding, speaking and writing were originally proposed. However, after discussion the competences for writing were omitted, as these were expected to be included elsewhere in the veterinary curriculum or to have been dealt with in previous primary and secondary education. One competence related to reading and understanding information was deleted.

In alignment with existing Competency Frameworks [5] and validated communication competences for Veterinarians [18], as well as Day One Competences [3], a set of ten communication competences relevant for veterinary undergraduate students was identified (see table 1 (Tab. 1)).

Even though communication is included in the respective European accreditation policy [3] and is already taught in many educational institutions, few standards are still available setting out how exactly this teaching should be implemented in highly subject-oriented curricula.

#### 3.2. Entrepreneurial competences

The EntreComp [20] was used as an essential framework for the initial definition of competences and checked for transferability and applicability in veterinary medicine. Furthermore, existing approaches to teach business and management skills were aligned [10], [11]. Hence, all 15 competences originating from the EntreComp were presented to experts and further discussed and refined. Nine competences were found to be sufficient for the veterinary curriculum (see table 2 (Tab. 2)).

In our model, there is no longer a division into different areas, since entrepreneurship is viewed as an area in itself. Some competences were combined, such as “taking the initiative” and “motivation and perseverance”, now referred to as motivation and determination (E1). In addition, “mobilising resources” and “mobilising others” from EntreComp became “Mobilising resources”, since personnel can also be seen as a resource.

While communication skills are explicitly mentioned within various frameworks, entrepreneurship is often associated only with business and economic skills, leaving out several more professional competences [7], [31], [34]. Draper and Uhlenhopp (2002) provided a very detailed curriculum for veterinary business systems, including teaching methods and examples of good practice [11]. They also discuss that curricula often leave very little room for new courses.

#### 3.3. Digital competences

Similar to the approaches within the competence area of entrepreneurship, the initial set of competences was based on DigComp [27], [28], and these were linked to the Day One Competences of the European System of Evaluation of Veterinary Training (ESEVT) [3] during the expert discussion. The initial total of 21 digital competences was reduced and refined into eight digital competences relevant for the veterinary education (see table 3 (Tab. 3)).

Digital skills are rarely addressed in the context of veterinary accreditation policies [3], [31], [34]. It is stated that access to resources, including digital files and resources for retrieving relevant information and literacy skills should be provided and trained. However, more detailed competences or learning outcomes are not defined. Within the standard operating procedures of ESEVT adapted in 2019, technology involved within the production and processes of animal feedstuffs and food hygiene are referred to [3]. Within the framework of the Association of American Veterinary Medical Colleges (AAVMC) information technology is grouped under professionalism and professional identity [7].

## Discussion

The SOFTVETS Competence model was derived after an iterative consultation process involving veterinary experts as well as experts in the areas of the addressed competences. The list of competences should provide an informative basis for educators in the fields of communication, entrepreneurship and digital literacy. The competences were adapted and defined for application in the context of veterinary medicine. This will ensure that a wide range of roles and professions that can be pursued in the professional life of a veterinarian are covered (e.g. as a practitioner, hygienist, scientist, national veterinary services officer, animal welfare officer, designated veterinarian, etc.). The transfer of knowledge and skills should not only relate to undergraduate education. Non-technical skills as well as veterinary clinical skills should be further developed in the context of continuing professional education.

Within the competence framework of Bok et al. (2011), communication as well as entrepreneurial competences are included. However, digital competences are not addressed explicitly [5]. In Bok’s framework two competences are for the area of communication and four competences are postulated within the domain of entrepreneurship [5]. These competences definitions are rather broad and do not adhere to the five quality criteria applied within this project [29], [30]. Within the SOFTVETS competence model, the competences are defined more specifically and with the aim of being individually assessed.

During the expert discussions, it was noticed that communication is taught at various stages during the veterinary undergraduate study. However, this depends heavily on elective courses. Anchoring thereof in the curriculum internationally has not yet been implemented. Using the SOFTVETS competence model, it is not necessary to implement communication training as a new subject in itself. Preferably, the model can rather be used to identify important competence areas addressing cognitive and practical skills, which may be trained as part of other subjects. 

The SOFTVETS competence model explicitly lists digital skills specifically relevant to the veterinary field of study. Today, technology is used in a diverse number of areas. Therefore, the focus should not only be on certain subject areas and specialisations, but each student should also acquire basic digital knowledge and skills.

Entrepreneurship refers to a broader context and does not only include business management skills. As Draper and Uhlenhopp (2002) underline that the importance of a successful veterinary career should be the motivating incentive and starting point for curriculum change [11], the SOFTVETS competence model provides an overview of topics to be covered. In this model, not only the issues of business management were taken into account, but also other entrepreneurial perspectives from inside and outside the veterinary profession were considered.

As already stated above, the three competence areas are closely interlinked. The total number of 27 competences are also interrelated and interconnected. We are not suggesting that the learner should attain the highest level in all competences, or have the same proficiency for all competences. Rather, this competence model should help educators in implementing and integrating key competence training within the curriculum. In the expert discussions, it became clear how diverse the transfer of competencies within veterinary education currently is, both nationally and internationally. This also requires different approaches and formats of integration. The implementation can either take place as stand-alone courses or be integrated into existing curricula. Higher education institutions already teach various vocational skills. This detailed list of competences in the three specific areas can then be used as a tool to better identify existing deficits and thus enable further individual development.

The widely used subject-oriented curricula also pose a major challenge. Attempts are being made to integrate competence-oriented teaching into this system, but of course it is reaching its limits. Another point of discussion was the number of hours to be spent on teaching key competences. All experts agreed that the teaching of non-technical skills should be closely linked to technical skills and should not be taught completely in isolation. This in turn means that lecturers from different fields should cooperate with one another. In addition to the need for qualified educators to enable networked teaching of technical and non-technical skills, the motivation and acceptance of students is also an important factor for the successful implementation of professional competence training. Henry and Treanor (2010) found that employers give much greater relevance and priority to business and entrepreneurial skills than students do during their undergraduate training [22]. They also point out that the challenge to reduce the discrepancy between employers’ and students’ perceptions of the value of business and entrepreneurial education should be addressed and student-centred innovative pedagogical approaches are important.

In addition to the literature, experts from the participating universities were involved in the discussion within the framework of the funded SOFTVETS project This represents a certain limitation. Other experts would certainly have provided even more input. This limitation could now be minimised by publishing the current proposal for further consideration and discussion by other stakeholders.

## Conclusion

This article describes the collaborative development process of the first work package within the SOFTVETS project and presents the competence model as an outcome. In the next work packages, learning objectives as well as teaching and assessment methods will be collected for each defined competence. Training concepts on how to train facilitators to provide competence training in the three areas were developed and tested in a pilot project in February 2020. The competence model has not yet been adapted to or tested in real settings. In addition to the learning and teaching instruments, a toolkit for evaluating the teaching of the above mentioned competences will be developed in order to standardise the implementation as well as the evaluation and assessment of competence training.

## Supportive information

All resources are available online: http://www.softvets.eu

## Acknowledgements

We would like to thank all experts involved in the development of the SOFTVETS competence model: Juraj Grizelj, Daniel Labas, Danijela Horvatek Tomic, Marina Pavlak, Tanja Knific, Marc Drillich, Birgit Hladschik-Kermer, Ingrid Preusche, Metka Kuhar, Istvan Toth, Tibor Bartha, Alen Slavica, Ton Willemse, Phillip Duffus, Claire Vinten.

Furthermore, we wish to thank the students from the Standing Committee on Veterinary Education (SCoVE) of the International Veterinary Students Association (IVSA) as well as those members from The European Association of Establishments for Veterinary Education [EAEVE) who were involved in the project.

## Funding

The project was funded by the Erasmus+ Programme of the European Union.

Key Action: Cooperation for innovation and the exchange of good practices

Action Type: Strategic Partnerships for higher education

Project Reference: 2018-1-HR01-KA203-047494

## Profiles

**Name of the institution:** University of Veterinary Medicine Hannover, Foundation

**Degree: **Veterinary medicine 

**Number of students per semester:** 250

**Has a longitudinal communication curriculum been implemented?** Partially

**In which semesters are communication and social skills taught?** 1, 2, 9 or 10 (compulsory); 1-4, 5-8 (optional extracurricular classes)

**Which teaching formats are used?**

- Compulsory: Lectures, Exercises in small groups including conversation simulation with actors and feedback
- Optional: Seminars with exercises in small groups including conversation simulation with actors and feedback, E-Learning

**In which semesters are communication and social skills examined (formative or relevant to degree progression and/or graded?)** Formative, all semesters, relevant to degree progression in the 9th semester

**Which examination formats are used?**

- formative as feedback,
- electronic objective structured clinical examinations (eOSCE)

**Which (Clinic, Institute) oversees the development and implementation?** Centre for E-Learning, Didactics and Educational Research, Small Animal Hospital

## Current professional roles of the authors

- Christin Kleinsorgen is research assistant in the e-learning department. She develops and advises on the creation of digital teaching and learning materials, teaches communication and researches about e-learning and professional skills development. She is actively involved in design and implementation of communication training in the Skills Lab.
- Evelyn Steinberg is a researcher at Vetmeduni Vienna focusing on teaching and learning in competence-based medical education. She studied psychology and specialized on education and evaluation.
- Rudolf Dömötör is Managing Director of WU Vienna Entrepreneurship Center and of the Entrepreneurship Center Network (ECN), a joint initiative of six Viennese universities promoting cross-disciplinary entrepreneurship among students and faculty. He is a co-founder of Entrepreneurship Avenue, the largest entrepreneurship event series with a focus on students in Europe.
- Jelka Zabavnik Piano is teaching Genetics and Methodology of scientific research work to veterinary students. She is in charge of the quality of higher education at home institution. Her research is focused on the use of information on genetic variability to detect disease susceptibility and to identify animal species.
- Jože Rugelj is professor and head of the Chair of Didactics of computer science at the Faculty of Education, University of Ljubljana. His main research areas are educational technology and innovative learning approaches with ICT with a special focus on game design based learning.
- Mira Mandoki is associate professor and the head of Pathology at the University of Veterinary Medicine Budapest, Hungary. She was involved in educational research from 2010. She participated in four EU projects in different consortia. She is in charge for the local teacher training at her university.
- Lada Radin’s ongoing position is at the Faculty of Veterinary Medicine, Zagreb, as a head of EU funds office. Previously a teacher in Veterinary Physiology with scientific interest in small ruminants. She is lately invested in higher education policies as well as tools & techniques of modern higher education.

## Competing interests

The authors declare that they have no competing interests. 

## Figures and Tables

**Table 1:**
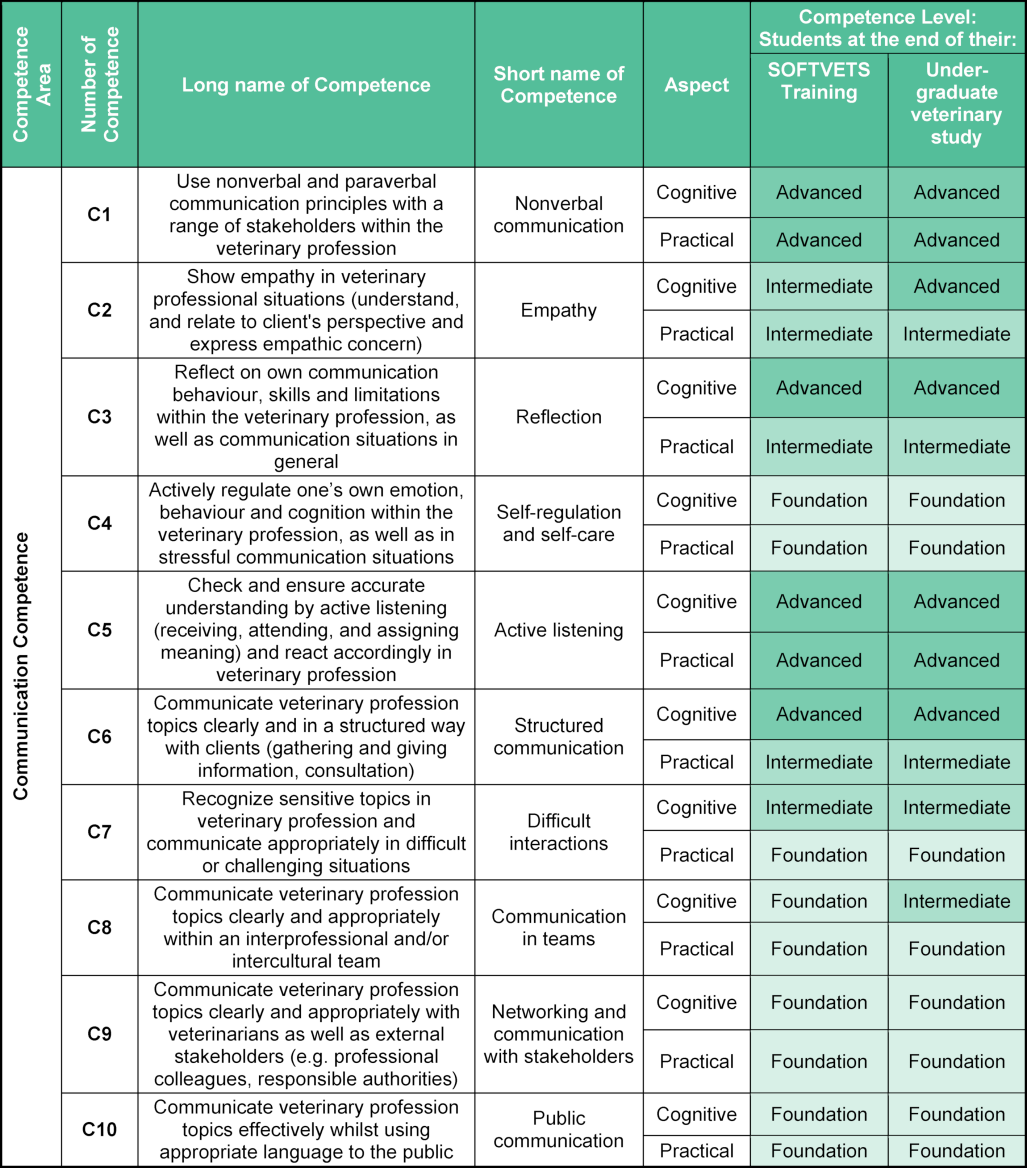
SOFTVETS communication competences

**Table 2:**
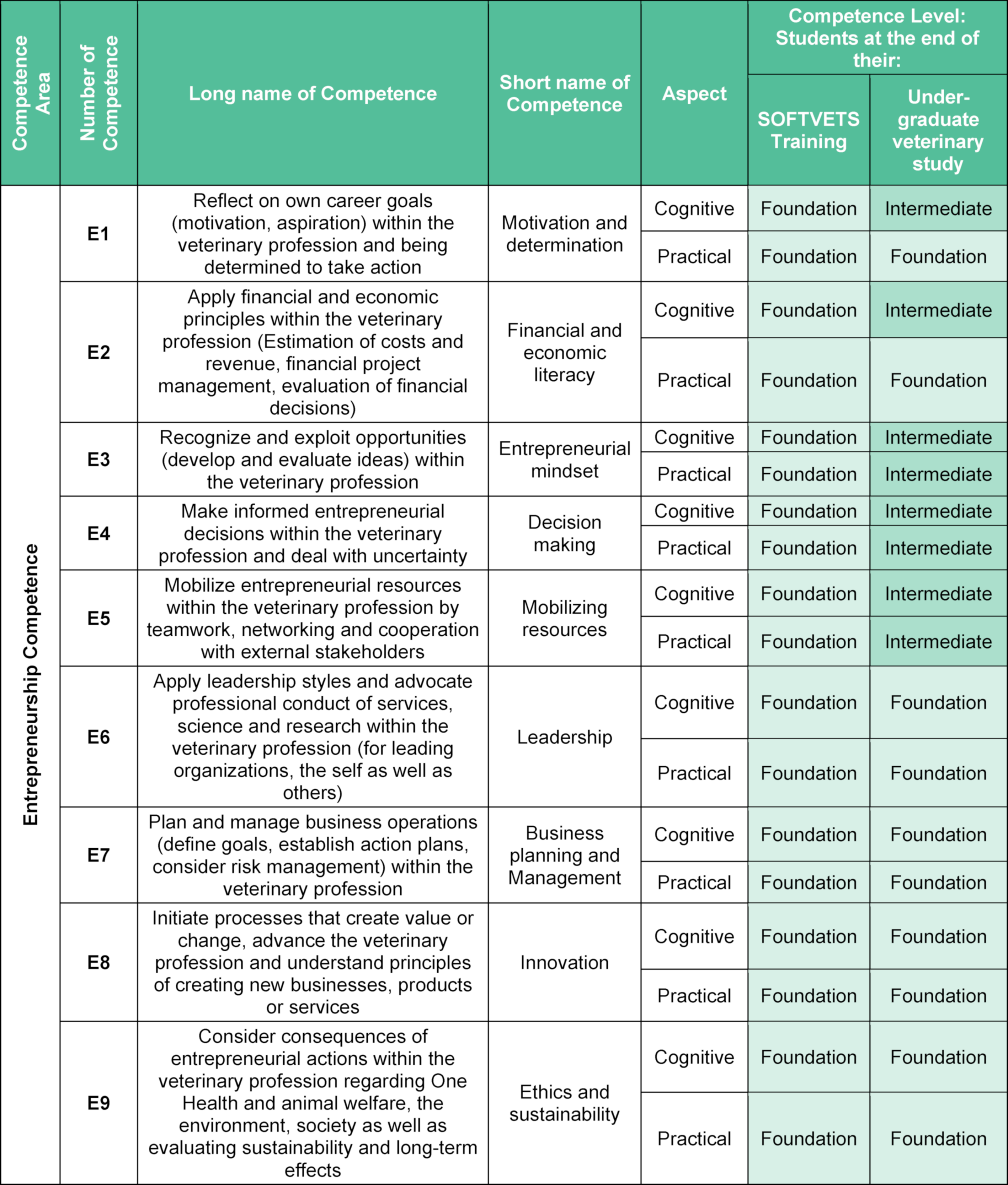
SOFTVETS entrepreneurial competences

**Table 3:**
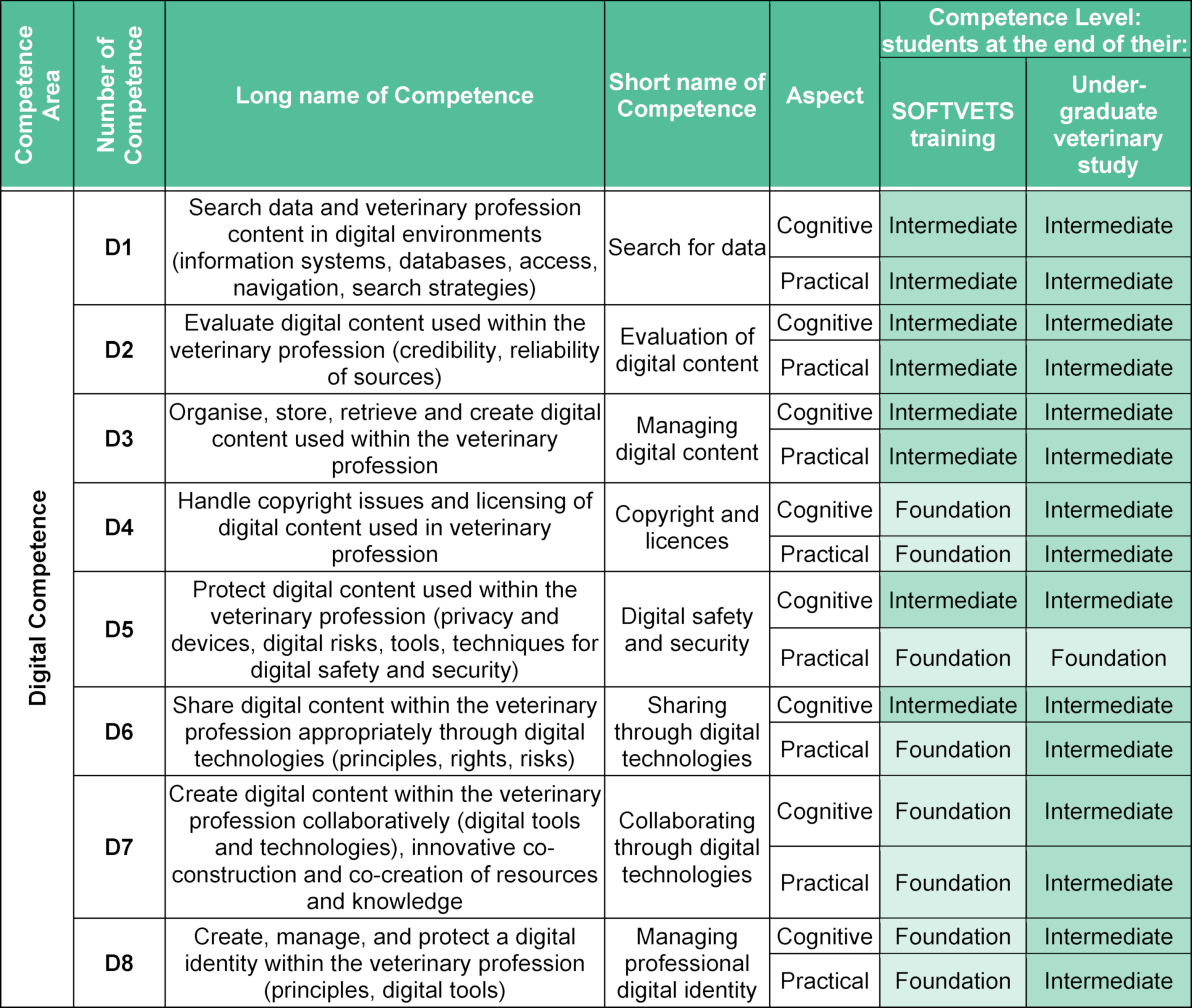
SOFTVETS digital competences
